# Effect of the COVID-19 Pandemic on the Management of Breast Cancer Patients

**DOI:** 10.3390/jcm13247673

**Published:** 2024-12-16

**Authors:** Yasin Dalda, Sami Akbulut, Zeynep Kucukakcali, Zeki Ogut, Ozlem Dalda, Saadet Alan, Burak Isik

**Affiliations:** 1 Department of Surgery and Liver Transplant Institute, Inonu University Faculty of Medicine, Malatya 44280, Turkey; 2 Department of Biostatistics and Medical Informatics, Inonu University Faculty of Medicine, Malatya 44280, Turkey; 3 Department of Pathology, Inonu University Faculty of Medicine, Malatya 44280, Turkey

**Keywords:** COVID-19 pandemic, breast cancer, mastectomy, breast-conserving surgery, neoadjuvant chemotherapy, delayed surgery

## Abstract

**Background:** The COVID-19 pandemic has significantly affected breast cancer patients by causing delays in diagnosis and treatment processes. This study aims to investigate the effects of the pandemic on the treatment process and short-term outcomes of breast cancer patients. **Methods:** This retrospective, cross-sectional, single-center study included 414 patients who underwent surgery for breast cancer at the Inonu University General Surgery Clinic between March 2018 and June 2021. The patients were divided into two groups: pre-pandemic (Pre-COVID-19; n = 240) and pandemic (COVID-19 Era; n = 174) periods. The groups were compared in terms of demographic, clinical, and histopathological variables. **Results:** During the pandemic period, the use of neoadjuvant therapy (from 21.3% to 34.5%) and preoperative PET-CT imaging (from 80.4% to 90.8%) rates increased, while breast-conserving surgery (from 27.9% to 19.0%) and the presence of comorbid diseases (from 45.0% to 29.9%) decreased significantly. While there was no significant difference between the groups in terms of the time from diagnosis to surgery (25 vs. 28.5 days, *p* = 0.121), the time to report the pathology result after surgery decreased during the pandemic period (28 vs. 23 days, *p* < 0.001). There was no significant difference between the groups in terms of immunohistochemical (ER, PR, Ki-67, E-cadherin, and c-erbB2), histopathological (lymphovascular invasion, perineural invasion, comedo necrosis, modified Bloom–Richardson grade, and TNM classification), and clinical (recurrence, metastasis, and axillary lymph node metastasis) features of the tumor. The mortality rate in the Pre-COVID-19 group (7.1%) was significantly higher than in the COVID-19 Era group (2.3%) (*p* = 0.049). Finally, in terms of the survival analysis, a statistically significant difference was found between the Pre-COVID-19 and COVID-19 Era groups in terms of the mean follow-up duration of the patients (*p* = 0.044). **Conclusions:** The study results show that the use of neoadjuvant therapy and radical surgery preference increased in breast cancer treatment during the pandemic period, but there was no significant change in tumor biology and histopathological features. Breast-conserving surgery rates, comorbidity rates, and pathology reporting times were significantly shortened. Long-term follow-up periods of 3 and 5 years are needed to see the impact of the pandemic on breast cancer patients.

## 1. Introduction

Severe Acute Respiratory Distress Syndrome Coronavirus-2 (SARS-CoV-2), the virus responsible for COVID-19, was first detected in December 2019 in Wuhan, China, and quickly escalated into a Public Health Emergency of International Concern (PHEIC) [1,2]. As a result, the World Health Organization (WHO) declared the COVID-19 outbreak a pandemic on 11 March 2020 [1]. Following the declaration of the pandemic and the increase in case numbers, countries closed their borders to international travel, imposed lockdowns, and implemented measures, such as social distancing and mask-wearing, to reduce transmission. With the rising number of cases, healthcare systems were placed under unprecedented strain. The first report published in August 2020 regarding the sustainability of essential health services showed that 90% of countries worldwide had been affected by the disruption of these services [3]. As the number of cases grew, hospital capacities and the shortage of healthcare workers impacted many countries, prompting radical measures to be taken.

In parallel with the detection of the first COVID-19 case in Turkey on 11 March 2020, similar measures were taken, and all non-emergency surgical procedures, including cancer surgeries, were postponed [4,5,6]. The implementation of strict policies to prevent the spread of COVID-19 infection caused delays in the management of non-COVID-19 health issues. One of these issues, the delays in cancer patient care, was recognized, and articles were published with recommendations on how to safely treat these patients [7,8,9]. It is a well-known fact that delays in the management of breast cancer, which is likely to occur in one in eight women over their lifetime, accounts for one in every four cancer cases and causes one in every six cancer-related deaths, would pose a serious public health problem on a global scale [10,11].

Breast cancer is a major cause of cancer-related deaths and is the most commonly diagnosed malignancy among women worldwide [12]. While the 5-year survival rate can reach up to 99% in early stages, it drops to 26% in patients diagnosed at advanced stages [13]. The COVID-19 pandemic, which has severely impacted the healthcare sector, has also affected the screening, diagnosis, and treatment processes for breast cancer patients. In many countries, screening programs were suspended, and changes were made in the management of treatment processes. As operating rooms were closed, there was an increase in the use of neoadjuvant therapy. Data from several countries on screening showed a decrease in breast cancer diagnoses in 2020 and delays in the time from diagnosis to surgery [14,15,16,17,18]. Following this period, there has been a consensus in the literature that the pandemic has delayed diagnosis and treatment for breast cancer. Studies have predicted that these delays caused by the pandemic in breast cancer patients would result in an increase in the 5-year mortality rate by 7.9–9.6% [19].

The collapse of healthcare systems in many countries during the pandemic and the disruptions in the diagnosis and treatment of cancer patients due to both the pandemic and the shortage of healthcare workers have been confirmed by numerous scientific publications [20,21,22,23]. Based on these results, it is expected that there will be deterioration in long-term outcomes [19]. In this study, which was prepared based on these findings, the aim is to investigate the impact of the COVID-19 pandemic on the treatment process and long-term outcomes of breast cancer patients, and to compare the survival, demographic, clinical, and histopathological data of breast cancer patients who were treated during and before the pandemic.

## 2. Materials and Methods

### 2.1. Type, Duration, and Location of the Study

This retrospective, cross-sectional, and single-center study includes patients who underwent surgery for breast cancer at the Department of General Surgery, Inonu University Faculty of Medicine, between March 2018 and June 2021.

### 2.2. Determination of the Research Universe and Study Group

Based on the data from the patient information and management system (HBYS) used in our hospital, 414 patients who underwent surgery for breast cancer between March 2018 and June 2021 were included in the study. The 240 patients treated between March 2018 and March 2020 were included in the pre-COVID-19 group, referred to as the Pre-COVID-19 group. The 174 patients who underwent breast cancer surgery between March 2020 and June 2021 were included in the COVID-19 period group, referred to as the COVID-19 Era group.

### 2.3. Inclusion and Exclusion Criteria

This study included breast cancer patients who underwent surgical treatment at our center and continued their postoperative follow-up and treatment at our hospital. Patients who were diagnosed with breast cancer but not operated on, those who were operated on at other centers and received oncological treatment at our center, or those who were only referred to the pathology department of our hospital for histopathological evaluation were excluded from the study.

### 2.4. Definitions, Parameters, and Variables Used in the Study

Using the ENLIL data system, which has been in use at our hospital since 2009, demographic data, surgical findings, histopathological data, and postoperative clinical follow-ups of patients were retrospectively recorded in an Excel file. The time between the date of the biopsy report and the day the patient underwent surgery was defined as “from diagnosis to surgery.” The time between the surgery date and the date the permanent pathology report was written was defined as “from surgery to pathology report.” For patients who were still alive, the follow-up period was defined as the time between the surgery date and the date of their last outpatient clinic visit or the day contact was made by phone. For deceased patients, the follow-up period was defined as the time between the surgery date and the day the patient passed away.

Neoadjuvant treatment was used for locally advanced breast cancer, inflammatory breast cancer, high-risk HER2 receptor positivity, high-risk triple-negative breast cancer, clinically positive axillary lymph nodes, delays in elective surgery due to the pandemic or other circumstances, and to downstage large tumors to enable breast-conserving surgery (BCS) [24,25]. Based on parameters such as the patient’s age, personal preferences, presence of comorbidities, breast size, histopathological and radiological features of the tumor, and the status of the axilla, either BCS (quadrantectomy ± axillary dissection and lumpectomy ± axillary dissection) or mastectomy ± axillary lymph node dissection was performed [26]. For BCS, factors considered included the patient’s preference, absence of contraindications for radiotherapy, DCIS/Tis, T1-2 tumors, and a tumor size < 3.5 cm. Axillary lymph node dissection was performed if one of the following criteria was present: clinically positive axillary lymph nodes, occult breast cancer with positive axillary lymph nodes, previous neoadjuvant therapy, inflammatory/T3/T4 breast cancers, positive sentinel lymph node, and cases where a sentinel lymph node biopsy was not possible [27]. Tumor staging was performed based on clinical, radiological, and histopathological features in all patients. The American Joint Committee on Cancer (AJCC) Cancer Staging 8th Edition was used for the anatomical staging of breast cancer based on clinical and pathological evaluations [28,29]. The TNM (T: primary tumor size, N: nodal involvement, M: metastasis) staging method was employed in this system.

The following variables were examined for comparison in this study: age (years), gender (female, male), comorbidity (hypertension, diabetes mellitus, etc.), American Society of Anesthesiologists (ASA) score, time from diagnosis to surgery (days), time from surgery to pathology report (days), preoperative 18FDG-PET-CT (18F-fluorodeoxyglucose positron emission tomography/computed tomography), neoadjuvant chemotherapy (CT), adjuvant chemoradiotherapy (CRT), type of surgery (mastectomy, BCS), weight of the mastectomy specimen (grams), which breast (left, right, bilateral), which quadrant (lower inner, lower outer, upper inner, upper outer, retroareolar), multicentricity status, multifocality status, histopathological type of breast cancer (DCIS: ductal carcinoma in situ; IDC: invasive ductal carcinoma; ILC: invasive lobular carcinoma; LCIS: lobular carcinoma in situ; NET: neuroendocrine tumor; etc.), modified Bloom–Richardson (MBR) grading (1-2-3), skin involvement, nipple involvement, lymphovascular invasion, perineural invasion, comedo necrosis, tubule formation, nuclear pleomorphism, mitotic count, tumor size (mm), total axillary lymphadenopathy (LAP), positive axillary LAP, estrogen receptor (ER) expression, progesterone receptor (PR) expression, Cerb-B2 (HER2/neu) expression, E-cadherin expression, Ki-67 (MIB-1) proliferation index, TNM classification system, postoperative recurrence, postoperative metastasis, and outcomes (alive, mortality).

### 2.5. Study Protocol and Ethics Committee Approval

This study involving human participants followed the ethical standards of the institutional and national research committee, alongside those of the 1964 Helsinki Declaration and its later amendments or comparable ethical standards. Ethical approval was obtained from the Inonu University Institutional Review Board (IRB) for non-interventional studies (Approval no: 2023/4444). The strengthening the reporting of observational studies in epidemiology (STROBE) guidelines were utilized to assess the likelihood of bias and the overall study quality [30].

### 2.6. Statistical Analysis

The variables in the study were summarized using frequencies (percentages) for categorical variables and median values with a 95% confidence interval for numerical variables. The Kolmogorov–Smirnov test was applied to assess the normality of the data distribution. For data that did not show a normal distribution, non-parametric statistical tests were preferred. In the statistical analysis, appropriate tests were selected based on the type and distribution of the variables. The Mann–Whitney U test was used to examine differences between two independent groups, Fisher’s exact chi-square test, chi-square test with Yates correction, and Pearson chi-square test were used to assess the relationship between categorical variables where appropriate. Kaplan–Meier survival analysis was conducted to evaluate the effect of metastasis status and the Pre-COVID-19 and COVID-19 Era groups on survival. Differences in survival rates obtained from the Kaplan–Meier analysis were compared using the log-rank test. In the survival data analysis, within-group differences were evaluated for statistical significance, and the effects on survival durations for both groups were assessed in detail. The significance level for the statistical analysis was set at *p* < 0.05, and the results obtained at this level were considered statistically significant. All analyses were performed using IBM SPSS 25.0 software to ensure the reliability and accuracy of the data.

## 3. Results

### 3.1. Analysis of Breast Cancer Screening Data Based on Pre-COVID-19 and COVID-19 Era

Patients who applied to the hospital for the breast outpatient clinic (ICD codes: N63, N64, C50) and breast cancer screening program (mammography and ultrasonography) in the pre- and post-pandemic eras were screened through the hospital HBYS system. Accordingly, while the number of patients who applied for screening before the pandemic was 2835, the number of patients who applied during the pandemic period was determined as 2165, which indicates a 23.6% decrease in breast screenings during the pandemic period. Similarly, in the screening conducted using the mentioned ICD codes, the number of patients who applied to the breast outpatient clinic before the pandemic period was 4809, while the number of patients who applied during the pandemic period was determined as 3868, which indicates a 19.5% decrease in breast screenings during the pandemic period.

### 3.2. Analysis of Data from the Entire Study Group

The median age of the patients in the study is 50 (95%CI = 49–52) years. Descriptive information for the variables in the dataset is provided in Table 1. According to Table 1, 58% of the patient groups were in the Pre-COVID-19 period, while 42% were in the COVID-19 Era period. The gender distribution shows 99% female and 1% male. The most common histopathological diagnosis is invasive ductal carcinoma (IDC) with 70.1%, while other types are distributed at lower rates. The rates of skin and nipple involvement are 8.9% and 6.0%, respectively. The rates of lymphovascular and perineural invasion are high, at 61.0% and 23.2%, respectively. Comedo necrosis was observed in 23.0% of cases. The positive rates for estrogen receptor (ER) and progesterone receptor (PR) are high, at 83.4% and 72.5%, respectively, while Cerb-B2 positivity is recorded at 31.1%. In the TNM classification, the most common stages are IA (19.3%) and IIA (23.4%), and the postoperative recurrence and metastasis rates are 2.2% and 9.0%, respectively. As a result, 94.9% of the patients survived.

### 3.3. Comparison of Pre-COVID-19 and COVID-19 Era Groups

#### 3.3.1. Univariate Analysis

Table 2 compares some demographic and clinical quantitative parameters between the Pre-COVID19 and COVID-19 Era groups. The median age of the patients is similar in both groups, with 51 years (95% CI = 50–54) in the Pre-COVID-19 group and 50 years (95% CI = 49–54) in the COVID-19 Era group. The time from diagnosis to surgery is 25 days (95% CI = 21–31) in the Pre-COVID-19 group and 28.5 days (95% CI = 26–35) in the COVID-19 Era group, with no statistically significant difference found (*p* = 0.121). The time from surgery to pathology report is 28 days (95% CI = 28–30) in the Pre-COVID-19 group and 23 days (95% CI = 22–26) in the COVID-19 Era group, and this difference is statistically significant (*p*< 0.001). No statistically significant differences were found between the groups for total specimen weight (*p* = 0.125), tubule formation (*p* = 0.156), nuclear pleomorphism (*p* = 0.230), and mitotic count (*p* = 0.681). The median tumor size is 24 mm, which is similar for both periods (*p* = 0.501). No significant differences were found for total (*p* = 0.016) and positive lymphadenopathy (LAP) counts (*p* = 0.793). The Ki-67 proliferation index is 20% in both the Pre-COVID-19 and COVID-19 Era groups, and there is no statistically significant difference (*p* = 0.059).

Table 3 compares some demographic and clinical qualitative data between the Pre-COVID-19 and COVID-19 Era groups. The comorbidity rate is 45% in the Pre-COVID-19 group and 29.9% in the COVID-19 Era group, with this difference found to be statistically significant (*p* = 0.002). Preoperative PET-CT usage is significantly higher in the COVID-19 Era group (80.4% vs. 90.8%; *p* = 0.006). Neoadjuvant therapy application is higher in the COVID-19 Era period as expected (21.3% vs. 34.5%; *p* = 0.003). Radical surgery was preferred more in the COVID-19 Era period (72.1% vs. 81.0%; *p* = 0.036). There was no significant relationship between the development of cancer in the right or left breast between the groups (*p* = 0.248). The lymphovascular invasion rate was found to be lower in the COVID-19 Era group, although this was not statistically significant (*p* = 0.061). ER (*p* = 0.842) and PR (*p* = 0.857) positivity rates are similar in both groups. The Cerb-B2 positivity rate significantly decreased in the COVID-19 Era group, although this did not reach statistical significance (*p* = 0.072). No significant changes were observed in the TNM classification (*p* = 0.448). Local recurrence (*p* = 0.312) and distant metastasis (*p* = 0.070) rates decreased in the COVID-19 Era period, but this did not reach statistical significance. While the follow-up duration is naturally shorter in the COVID-19 Era period, the mortality rate during follow-up was found to be lower in the COVID-19 Era period (*p* = 0.049).

#### 3.3.2. Multivariate Analysis

Multivariate analysis was performed considering the univariate analysis results (*p* < 0.05) of the Pre-COVID-19 and COVID-19 Era groups. Logistic regression analysis revealed that that there was a statistically significant relationship between independent variables, such as short time from surgery to the pathology report [*p* < 0.001; OR = 1.04 (95%CI = 1.02–1.06)], lower comorbidity [*p* = 0.003; OR = 1.95 (95%CI = 1.26–3.01)], increased neoadjuvant CT [*p* = 0.014; OR = 2.26 (95%CI = 1.18–4.32)], increased mastectomy [*p* = 0.020; OR = 1.85 (95%CI = 1.10–3.12)], low mortality [*p* = 0.032; OR = 1.12 (95%CI = 1.12–11.9)] and COVID-19 era.

### 3.4. Comparison of Surviving and Non-Surviving Patients with Brest Cancer

#### 3.4.1. Univariate Analysis

In Table 4, the patients are divided into two groups: those who are alive (alive group; n = 393) and those who have died (mortality group; n = 21), and the groups were compared for some quantitative variables. Twenty-one patients included in this study died due to primary breast cancer and related complications. None of the mortalities were secondary to COVID-19 infection. The median age of the alive group during the study period was 50 years (95% CI = 49–52), while the median age of the mortality group was 54 years (95% CI = 51–65). The time from diagnosis to surgery was 26 days (95% CI = 22–29) in the alive group, while it was 148 days (95% CI = 68–229) in the mortality group, and this difference was statistically significant (*p* = 0.001). The time from surgery to the pathology report was longer in the mortality group (26 vs. 33 days; *p* = 0.018). The specimen weight was 882 g (95% CI = 824–940) in the alive group and 1414 g (95% CI = 947–1876) in the mortality group, and the difference was significant (*p* = 0.001). Nuclear pleomorphism (*p* = 0.033) and mitotic count (*p* = 0.020) were higher in the mortality group. The median tumor size was 23 mm (95% CI = 22–25) in the alive group, while it was 42 mm (95% CI = 36–85) in the mortality group (*p* < 0.001). As expected, the median number of positive axillary lymph nodes (*p* = 0.032) and Ki-67 proliferation index (*p* = 0.009) were significantly higher in the mortality group.

In Table 5, the alive (n = 393) and mortality (n = 21) groups are compared for some categorical variables. Among the patients in the mortality group, 81.0% were from the Pre-COVID-19 period, while 19.1% were from the COVID-19 Era period, and this difference was statistically significant (*p* = 0.049). The presence of comorbidities was higher in the mortality group, but it did not reach statistical significance (*p* = 0.273). As expected, the ASA scores were higher in the mortality group (*p* = 0.022). A higher rate of neoadjuvant therapy was observed in the mortality group, which suggests that the tumors in these patients were at more advanced stages (24.9% vs. 61.9%; *p* = 0.001). All patients in the mortality group underwent radical surgery (74.6% vs. 100%; *p* = 0.017). Multicentricity was more common in the mortality group (10.2% vs. 52.4%; *p* < 0.001), while there was no significant difference between the groups regarding multifocality (*p* = 0.500). Nipple (5.1% vs. 23.8%; *p* = 0.006) and skin involvement (7.6% vs. 33.3%; *p* = 0.001) were significantly higher in the mortality group, and these differences were statistically significant. Lymphovascular invasion (59.4% vs. 90.5%; *p* = 0.009) and perineural invasion (21.9% vs. 47.6%; *p* = 0.014) were significantly higher in the mortality group. PR receptor positivity (73.7% vs. 47.4%; *p* = 0.025) was lower in the mortality group, while ER positivity (*p* = 0.136) was similar between the groups. There was a statistically significant difference in TNM classification between the alive and mortality groups (*p* < 0.001). Patients in the mortality group were more likely to be in stages IIIC and IV. As expected, local recurrence (1.3% vs. 20.0%; *p* < 0.01) and metastasis (5.1% vs. 85.0%; *p* < 0.001) rates were significantly higher in the mortality group.

#### 3.4.2. Multivariate Analysis

Multivariate analysis was performed considering the univariate analysis results (*p* < 0.05) of the Pre-COVID-19 and COVID-19 Era groups. Logistic regression analysis revealed that there was a significant relationship between the Ki 67 proliferation index [*p* < 0.38; OR = (95%CI = 1.10–1.20)], positive LAP [*p* < 0.044; OR = 1.27 (95%CI = 1.01–1.61)], and postoperative metastasis [*p* = 0.004; OR = 386 (95%CI = 11–13,183)] and mortality. On the other hand, it has been observed that the COVID-19 Era is not an independent factor affecting mortality.

### 3.5. Comparison of the Survival of Pre-COVID-19 and COVID-19 Era Groups

**Figure 1:**
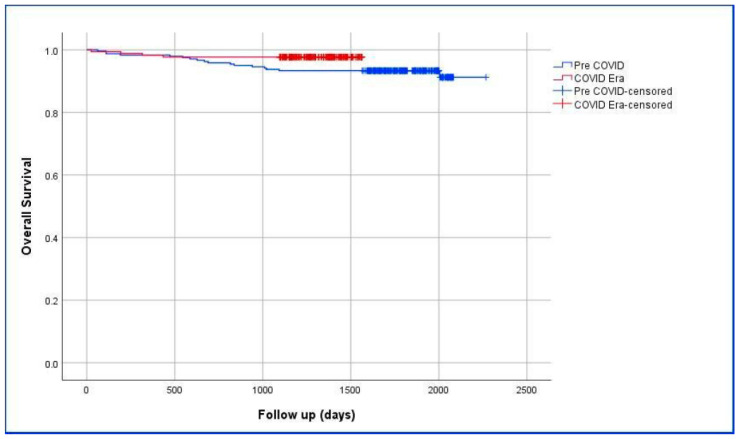
Kaplan–Meier estimate of overall survival for Pre-COVID-19 and COVID-19 Era groups.

The overall survival analysis of patients who underwent surgery in the Pre-COVID-19 and COVID-19 Era periods is presented in Table 6. A statistically significant difference was found between the groups in terms of the mean follow-up time (*p* = 0.044). The 1-, 3-, and 5-year survival rates of patients in the Pre-COVID-19 period were calculated as 98.3%, 93.3%, and 93.3%, respectively. The 1- and 3-year survival rates of patients in the COVID-19 period were calculated as 98.3% and 93.3%, respectively. The time required for 5-year survival results of patients in the COVID-19 period has not yet completed. Survival analysis based on the Kaplan–Meier estimate is shown in Figure 1.

## 4. Discussion

The COVID-19 pandemic, which affected millions of people worldwide, has severely impacted the healthcare systems of many countries. These effects have left negative traces not only in the diagnosis and treatment of infected patients, but also in cancer patients due to delays in diagnosis and treatment. The management of COVID-19-infected patients and the measures taken to reduce transmission had indirect effects, such as reducing hospital visits, disruptions in cancer patient referral chains, closure of operating rooms, and cancellation of elective surgeries [31,32]. As a result of these factors, delays in the diagnosis and treatment of cancer patients, especially during the active phases of the pandemic, have emerged. It has been shown that breast cancer patients experienced increased levels of anxiety and depression due to treatment delays and uncertainties surrounding their care [33,34]. The psychosocial impact of these disruptions has been profound, with many patients reporting feelings of isolation and distress due to the lack of support services and follow-up care [35]. Psychological burden has been particularly severe among newly diagnosed patients who have encountered additional stress factors related to cancer diagnoses amid a global health crisis [8]. Moreover, the pandemic has exacerbated existing inequalities in access to healthcare. Tsapatsaris et al. [36] emphasized that the barriers to breast cancer screening during the pandemic worsened, especially for marginalized communities. This inequality in access to care could have long-term effects on breast cancer outcomes, as delays in diagnosis and treatment are more prominent in lower-income groups and vulnerable populations [37]. The overall decrease in screening rates is concerning, with some regions reporting a reduction of up to 41% in breast cancer screening tests during the pandemic [38]. Since this study does not address changes in breast cancer screening programs for women, we do not have objective data on disruptions in screening programs at our center during the pandemic. However, studies conducted in Turkey have highlighted significant disruptions in screening programs and the detection of new breast cancer cases [39,40,41,42,43].

Breast cancer, a significant public health issue due to its incidence and related mortality, accounts for 24% of all cancers diagnosed and 15% of cancer-related deaths globally [44]. Approximately one in eight women is at risk of developing breast cancer. One of the most important factors in improving the success rates of breast cancer treatment and reducing mortality is the proper use of screening programs and early diagnosis. During the pandemic, these programs were disrupted. The lack of personal protective equipment, hospital capacities being focused on COVID-19 patients, and the need to protect both healthcare personnel and patients from transmission led to the suspension of elective imaging and procedures, including cancer screenings. In March 2020, the American Society of Breast Surgeons, American College of Radiology, and Society of Breast Imaging published a statement recommending the postponement of all breast screenings [45]. As a result, several studies have reported a decrease in screening mammograms [21,46]. A study evaluating the National Cancer Database in the United States showed a 12.4% decline in the expected overall cancer diagnoses in 2020 and 34,000 fewer new breast cancer diagnoses [47]. Although screening programs returned to normal in the following months, these disruptions led to expectations that cancer patients would present with more advanced stages. Mixed results have been reported in studies related to this. In an April 2021 study conducted in Italy, no differences were observed in tumor biology, but there was an increase in stage III disease and axillary node positivity [48]. Another multicenter study reported higher histological grade and axillary lymph node metastasis in patients operated during the pandemic [49]. A retrospective study comparing Pre-COVID-19 and COVID-19 periods showed no differences in breast cancer stage and tumor biology [50]. More recently, a multicenter study found a slight increase in tumor size and axillary node positivity [51]. Additionally, Bonadio et al. [52] emphasized that the negative impact of the pandemic on cancer treatment was primarily due to the reduction in screening and diagnostic procedures, which contributed to a shift in the cancer stage at diagnosis. Upon detailed examination of Table 1 and Table 2 in the present study, it is evident that the pandemic had no impact on disease stage, axillary node positivity, or tumor biology-related parameters. The most interesting aspect of this study, which differentiates it from many studies in the literature, is that the time between diagnosis and surgery during the pandemic was similar to the pre-pandemic period, and the pathology reports were approved in a shorter time than expected. This situation can be explained by several arguments. First, cancer patients were not ignored, even in the early phases of the pandemic, when fear of transmission and death was at its highest. Second, the reduced workload in pathology laboratories due to the suspension of non-cancer elective surgeries may have led to faster report preparation. Third, the isolation of the pathology laboratory from patients and their relatives likely led to a lower fear of disease transmission among the laboratory staff.

An analysis of the patient numbers in the present study reveals that there was no difference in the number of patients between the Pre-COVID-19 and COVID-19 Era periods. In other words, there was no decline in breast cancer surgeries during the pandemic at our center. This can be explained by two arguments. First, the other hospitals in our city were specialized in treating COVID-19 patients, and cancer patients were directed to our center, which may have prevented a decrease in patient numbers. Second, the fear of contracting cancer and the delays in treatment outweighed the concerns regarding the pandemic, causing patients to adhere to their diagnostic and treatment processes. Another clear finding of this study is that the comorbidity rates of patients during the pandemic were statistically significantly lower. Studies indicating that COVID-19 infections have a severe course in patients with comorbidities (such as diabetes mellitus, cardiovascular disease, pulmonary disease, etc.) might have caused a significant fear of death in this patient group. Additionally, factors such as government-imposed lockdowns for this group of patients may have contributed to a decrease in hospital visits.

During the pandemic, the Breast Cancer Consortium published a strategy recommendation consisting of three main levels (A, B, C) based on cancer and related symptoms, outlining how multidisciplinary breast cancer treatment should be safely carried out [9]. According to this strategy, triple-negative breast cancer, c-erbB2 (+) breast cancers, inflammatory breast cancers, stage I or II ER (+)/HER2 (−) breast cancers, stage I c-erbB2 (+) breast cancers, and ER (+) patients with a DCIS were classified under level B. These patients were identified as those for whom treatment should begin before the end of the pandemic [9]. In fact, the article’s text states that most breast cancers belong to level B, and it was recommended that treatment for these patients be delayed until surgical conditions were met, with the initiation of neoadjuvant therapy being appropriate. This strategy was globally accepted, and during the pandemic, there was a significant increase in the use of neoadjuvant therapy until the safe application of surgical procedures [53]. A retrospective study conducted in Turkey by Koca and Yildirim [43] showed an increase in the use of neoadjuvant therapy, axillary involvement, and tumor size during the pandemic. Similarly, in a study by Hawrot et al. [54], an increase in the shift toward neoadjuvant therapy and the detection of more advanced-stage tumors during the pandemic were observed, similar to the Turkish study. Vanni et al. [55] reported that the pandemic caused delays in diagnosis and treatment, and many patients required adjuvant therapy due to advanced cancer stages at diagnosis [49,55]. In the present study, the use of neoadjuvant therapy during the pandemic increased by 1.95 (95% CI = 1.26–3.03) times. When Table 2 and Table 3 are evaluated together, it is seen that this increase is not related to tumor biology and stage, but is more related to the approaches recommended in national and international breast cancer consensus meetings [9,56].

During the pandemic, there were also changes in the surgical treatment of breast cancer. In patients eligible for BCS, mastectomy was avoided to shorten the duration of surgery, and for patients undergoing mastectomy, reconstructive procedures were strictly avoided to reduce both duration and complication risks [53,57,58]. Ilgun and Ozmen [59] noted that many reconstructive procedures were postponed to optimize healthcare resources, which could have long-term effects on patient outcomes. However, there are also studies in the literature showing that the options for mastectomy and BCS did not change during the pandemic [60,61]. In the present study, during the pandemic, the rate of mastectomy increased 1.65 times (95% CI = 1.03–2.65) compared to BCS, which is consistent with some studies in the literature [62,63]. One of the key reasons for the decreased preference for BCS during the pandemic may be that patients avoided frequent hospital visits for postoperative radiotherapy [64]. When subgroup analyses were performed, the neoadjuvant therapy (*p* < 0.001), metastasis (*p* = 0.029), and mortality (*p* = 0.017) rates in patients undergoing mastectomy were found to be 4.9 (95% CI = 2.4–10.0), 3.9 (95% CI = 1.2–13.1), and 7.6 times (95% CI = 1–56.7) higher, respectively, compared to the BCS group. These results are an indirect indication that even patients who received neoadjuvant therapy placed more trust in options like mastectomy, which completely removes the breast tissue. In the study by Gentile et al. [65] comparing patients who underwent BCS and mastectomy after neoadjuvant therapy, distant recurrence-free survival (*p* = 0.031) and event-free survival (*p* = 0.034) were better in the BCS group than in the mastectomy group. The study’s data indicated that patients who received a BCS exhibited better overall survival (*p* = 0.010) and distant metastasis-free survival (*p* = 0.015) outcomes.

The most important parameters related to the management of breast cancer and the success of the treatment methods used are distant metastasis, local recurrence, and disease-free survival and overall survival. The long-term effect of the pandemic on survival has not yet been clearly established in the literature. To statistically demonstrate the impact of the pandemic on survival, either prospective projections should be made, or data from 1-, 3-, and 5-year follow-ups should be analyzed and compared with the pre-pandemic period. When the data of the present study are examined, the 1-year mortality ratio in patients who underwent breast cancer surgery during the pandemic was found to be 3.24 times (95% CI = 1.07–9.80) lower than pre-pandemic era. Upon reviewing Graphic-1, a survival curve in favor of the COVID-19 Era group is observed. Therefore, it is not expected that one-year mortality ratio will change in the long-term projections regarding the impact of the COVID-19 pandemic on breast cancer that will be planned for the future. In our opinion, stronger conclusions should be drawn after waiting for 5-year follow-ups. In the COVID-19 Era group, recurrence and metastasis rates are lower, but this difference did not reach statistical significance.

In the studies conducted in the literature, there is a general consensus that diagnosis and treatment of breast cancer patients were delayed during the pandemic, and it is anticipated that long-term outcomes (metastasis, recurrence, and survival) will worsen. While there have been many opinions in the literature regarding the impact of the time between diagnosis and treatment initiation on prognosis, a consensus has not yet been reached [66,67]. Kothari et al. [67] have suggested that a delay of 3–6 months in starting treatment has an adverse effect on tumor size, stage, and prognosis. Bleicher [68] stated that patients with breast cancer should undergo surgical treatment within 90 days of diagnosis, chemotherapy within 120 days, and radiation therapy within 365 days for those receiving chemotherapy. According to a meta-analysis by Hanna et al. [69], delays of 8 and 12 weeks in starting surgical treatment for breast cancer increased mortality by 17% and 26%, respectively. In the study we present here, it has been shown that the pandemic did not cause delays in surgical treatment. Furthermore, it was shown that the permanent pathology results were reported earlier during the pandemic, which, to our knowledge, is a first in the literature.

There are a considerable number of studies in the literature suggesting that the pandemic period caused disruptions in breast cancer screening programs. We believe that it would be more appropriate to evaluate the results of meta-analysis in this regard. In a meta-analysis study analyzing data from 31 articles, Ng et al. [70] reported that they found a decrease of 41–53% and 18–29% in breast cancer screening and diagnosis rates, respectively, and stated that this decrease was more pronounced in countries that were quarantined. The authors stated that it was too early to comment on whether delayed screening during the pandemic increased breast cancer mortality. In a meta-analysis study analyzing data from 74 articles, Li et al. [71] reported that they found a decrease of ≥49% in breast cancer screening in more than half of the studies. The authors stated that they found a decrease of ≥25% in breast cancer diagnoses in two-thirds of the articles. When the data from the study presented here are analyzed, it is understood that the decreases in screening rates during the pandemic are consistent with the literature or even lower. According to a literature review covering 10 studies, Shah et al. [72] stated that, although there were disruptions in breast outpatient services and imaging during the pandemic, breast cancer surgeries were not significantly affected during the pandemic. When the groups of the study presented here are compared, it will be understood that there was no significant decrease in breast cancer surgery during the pandemic period, which is because our center is a reference center. However, a decrease in breast outpatient clinic visits was detected, similar to the authors’ conclusion. Budiarta et al. [73] prepared a systematic review consisting of 15 studies and the authors stated that there was an increase in simple mastectomy, BCS, waiting time, and time to start treatment when compared to the pre-pandemic period. On the other hand, they stated that they detected a decrease in the length of hospital stay and a tendency not to use radiotherapy or to use hypofractionated doses during the pandemic period.

This study has several limitations. Firstly, being a retrospective and single-center study is the main limitation that hinders generalizable conclusions. However, in the case of the pandemic, there is no alternative other than constructing a control group from retrospective data to compare with the pre-pandemic period. Secondly, the fact that this study was not designed as a multicenter study is another significant limitation; however, creating a consensus with another center that has a similar patient group is not as easy as expected under pandemic conditions. The third limitation of present study is the challenge in obtaining data concerning the interval between patients’ initial presentation and diagnosis. Finally, the 5-year survival rates of the patient cohort analyzed in this study will be re-analyzed in a future study and shared with readers as a new piece of research.

## 5. Lessons Learned and Future Perspective

Breast cancer, which has the highest incidence and mortality in women worldwide, and the COVID-19 pandemic, which has caused the death of millions of people, are two different clinical entities. But, it is a known fact that the pandemic causes disruptions in every stage of the management of breast cancer patients. Stay at home, social isolation, and use of personal protective equipment have been important for survival in immunocompromised patients, such as those with breast cancer [74,75]. However, delaying breast cancer screening during the pandemic to save healthcare resources and prevent patients from infection is unfeasible and will probably postpone the diagnosis and treatment of breast cancer [70]. In order to overcome possible pandemics with minimal casualties, it is important to develop some perspectives and health policies about the future. From the perspective of breast cancer, policies should be developed on issues such as spreading self-examination programs in the society and thus ensuring that cancer is detected at an early stage, developing telecommunication and internet networks to deliver the telemedicine system to the whole society, structuring hospitals specifically for pandemics, developing the home-care system, and carrying out multidisciplinary teams via teleconferencing [76,77,78]. In recent years, the use of artificial intelligence-based algorithms to detect and classify patients at risk of cancer is one of the most appropriate approaches in the diagnosis of patients at risk of breast cancer and the development of treatment protocols [79,80], and these algorithms can be further developed and easily used during the pandemic. Combining bra data, which is expected to be widely used in breast cancer screening, with artificial intelligence algorithms will be useful during pandemic periods [81,82,83].

## 6. Conclusions

When evaluated in conjunction with the literature, this study shows that the postponement of screening programs during the pandemic led to delays in diagnosis, the suspension of elective surgeries, and delayed initiation of treatment. As a result, patients received treatment at more advanced stages (e.g., tumor size, axillary lymph node involvement), which affected many cancer characteristics (recurrence, metastasis, mortality, etc.). However, the long-term outcomes regarding how the pandemic affected the survival of this group of patients have not yet been published. Specifically, in this study, it was found that the pandemic had no significant impact on important parameters, such as tumor biology (ER, PR, Ki-67, E-cadherin, and c-erbB2), histopathological features, or axillary lymph node metastasis. Due to the nature of the pandemic, a significant increase in neoadjuvant therapy usage was observed. On the other hand, preoperative PET-CT use, BCS options, and the time taken to report pathology results after surgery were significantly reduced.

## Figures and Tables

**Table 1:** Demographic and clinical features of entire cohort.

Parameters	Categories	Number (%)
Group	Pre-COVID-19	240 (58.0)
	COVID-19 Era	174 (42.0)
Gender	Female	410 (99.0)
	Male	4 (1.0)
Comorbidity	No	254 (61.3)
	Yes	160 (38.7)
ASA scores	1	83 (20.05)
	2	287 (69.3)
	3	44 (10.6)
Preoperative PET-CT	No	63 (15.2)
	Yes	351 (84.8)
Neoadjuvant CT	No	303 (73.2)
	Yes	111 (26.8)
Adjuvant CRT	No	53 (12.8)
	Yes	360 (87.2)
Type of surgery	Mastectomy	314 (75.9)
	BCS	100 (24.1)
Which breast	Right	197 (47.6)
	Left	217 (52.4)
Which quadrant	Lower outer	80 (19.3)
	Lower inner	47 (11.4)
	Upper outer	185 (44.7)
	Upper inner	88 (21.3)
	Retroareolar	14 (3.4)
Multicentricity	No	363 (87.7)
	Yes	51 (12.3)
Multifocality	No	360 (87.0)
	Yes	54 (13.04)
Histopathological diagnosis	Apocrine carcinoma	2 (0.5)
	DCIS	16 (3.9)
	IDC + ILC	10 (2.4)
	IDC	290 (70.1)
	ILC	29 (7.0)
	Micropapillary carcinoma	10 (2.4)
	Mucinous carcinoma	17 (4.1)
	Tubular carcinoma	2 (0.5)
	LCIS	2 (0.5)
	Malignant phyllodes tumor	1 (0.2)
	Medullary carcinoma	10 (2.4)
	Metaplastic carcinoma	5 (1.2)
	Microinvasive carcinoma	2 (0.5)
	NET (poorly differentiated)	7 (1.7)
	NET (well differentiated)	2 (0.5)
	Triple-negative breast carcinoma	9 (2.2)
MBR grade	1	39 (11.3)
	2	132 (38.4)
	3	173 (50.3)
Skin involvement	No	377 (91.1)
	Yes	37 (8.9)
Nipple involvement	No	389 (94.0)
	Yes	25 (6.0)
Lymphovascular invasion	No	161 (39.0)
	Yes	252 (61.0)
Perineural invasion	No	317 (76.8)
	Yes	96 (23.2)
Comedo necrosis	No	319 (77.1)
	Yes	95 (23.0)
ER	Negative	67 (16.6)
	Positive	336 (83.4)
PR	Negative	109 (27.5)
	Positive	287 (72.5)
c-erbB2	Negative	273 (68.9)
	Positive	123 (31.1)
E-cadherin	Negative	34 (13.6)
	Positive	216 (86.4)
TNM classification	0	18 (4.35)
	IA	80 (19.3)
	IIA	97 (23.4)
	IIB	55 (13.3)
	IIIA	65 (15.7)
	IIIB	34 (8.2)
	IIIC	48 (11.6)
	IV	17 (4.1)
Recurrence	No	401 (97.8)
	Yes	9 (2.2)
Metastasis	No	373 (91.0)
	Yes	37 (9.0)
Outcomes	Alive	393 (94.9)
	Mortality	21 (5.1)

COVID-19: coronavirus-19; PET: positron emission tomography; ASA: American Society of Anesthesiologists; CT: chemotherapy; CRT: chemoradiotherapy; DCIS: ductal carcinoma in situ; IDC: invasive ductal breast cancer; ILC: invasive lobular breast cancer; LCIS: lobular carcinoma in situ; NET: neuroendocrine tumor; MBR: modified Bloom–Richardson; ER: estrogen receptor; PR: progesterone receptor; TNM: primary tumor size (T), nodal involvement (N), M: metastasis (M).

**Table 2:** Comparison of Pre-COVID-19 and COVID-19 Era groups in terms of quantitative variables.

Parameters [Median (95%CI)]	Pre-COVID-19 (n = 240)	COVID-19 Era (n = 174)	*p*
Age (years)	51 (50-54)	50 (49-54)	0.857
From diagnosis to surgery (days)	25 (21-31)	28.5 (26-35)	0.121
From surgery to pathology report (days)	28 (28-30)	23 (22-26)	<0.001
Specimen weight (gram)	876 (792-946)	939 (830-988)	0.125
Tubule formation	3 (3-3)	3 (3-3)	0.156
Nuclear pleomorphism	3 (3-3)	3 (3-3)	0.230
Mitotic Count	2 (2-3)	2(2-3)	0.681
Tumor size (mm)	24 (22-26)	24 (20-28)	0.501
Total LAP	8.5 (5-10)	10.5 (8-12)	0.116
Positive LAP	0 (0-0)	0 (0-0)	0.793
Ki-67 (%)	20 (20-30)	20 (20-25)	0.059

LAP: lymphadenopathy; Ki-67: proliferation index.

**Table 3:** Comparison of Pre-COVID-19 and COVID-19 Era groups in terms of qualitative variables.

Parameters	Categories	Pre-COVID-19 (n = 240)	COVID-19 Era (n = 174)	*p*
Gender	Female	237 (98.8)	173 (99.4)	0.642 *
	Male	3 (1.3)	1 (0.6)	
Comorbidity	No	132 (55.0)	122 (70.1)	0.002 **
	Yes	108 (45.0)	52 (29.9)	
ASA scores	1	47 (19.6)	36 (20.7)	0.716 **
	2	165 (68.8)	122 (70.1)	
	3	28 (11.7)	16 (9.2)	
Preoperative PET-CT	No	47 (19.6)	16 (9.2)	0.006 ***
	Yes	193 (80.4)	158 (90.8)	
Neoadjuvant CT	No	189 (78.8)	114 (65.5)	0.003 **
	Yes	51 (21.3)	60 (34.5)	
Adjuvant CRT	No	29 (12.1)	24 (13.8)	0.727 ***
	Yes	210 (87.9)	150 (86.2)	
Type of surgery	Mastectomy	173 (72.1)	141 (81.0)	0.036
	BCS	67 (27.9)	33 (19.0)	
Which breast	Right	120 (50.0)	77 (44.3)	0.248 **
	Left	120 (50.0)	97 (55.8)	
Which quadrant	Lower outer	46 (19.2)	34 (19.5)	0.091 **
	Lower inner	21 (8.8)	26 (14.9)	
	Upper outer	116 (48.3)	69 (39.7)	
	Upper inner	52 (21.7)	36 (20.7)	
	Retroareolar	5 (2.1)	9 (5.2)	
Multicentricity	No	214 (89.2)	149 (85.6)	0.280 **
	Yes	26 (10.8)	25 (14.4)	
Multifocality	No	204 (85.0)	156 (89.7)	0.215 ***
	Yes	36 (15.0)	18 (10.3)	
MBR grade	1	25(12.3)	14 (9.9)	0.613 ***
	2	74 (36.5)	58 (41.1)	
	3	104 (51.2)	69 (48.9)	
Skin involvement	No	220 (91.7)	157 (90.2)	0.740 ***
	Yes	20 (8.3)	17 (9.8)	
Nipple involvement	No	228 (95.0)	161 (92.5)	0.405 ***
	Yes	12 (5.0)	13 (7.5)	
Lymphovascular invasion	No	84 (35.2)	77 (44.3)	0.061 **
	Yes	155 (64.9)	97 (55.8)	
Perineural invasion	No	184 (77.0)	133 (76.4)	0.896 **
	Yes	55 (23.0)	41 (23.6)	
Comedo necrosis	No	186 (77.5)	133 (76.4)	0.800 **
	Yes	54 (22.5)	41 (23.6)	
ER	Negative	38 (16.3)	29 (17.1)	0.842 **
	Positive	195 (83.7)	141 (82.9)	
PR	Negative	63 (27.9)	46 (27.1)	0.857 **
	Positive	163 (72.1)	124 (72.9)	
c-erbB2	Negative	149 (65.4)	124 (73.8)	0.072 **
	Positive	79 (34.7)	44 (26.2)	
E-cadherin	Negative	19 (14.6)	15 (12.5)	0.762 ***
	Positive	111 (85.4)	105 (87.5)	
TNM classification	0	14 (5.8)	4 (2.3)	0.448 **
	1A	50 (20.8)	30 (17.2)	
	2A	57 (23.8)	40 (23.0)	
	2B	32 (13.3)	23 (13.2)	
	3A	32 (13.3)	33 (19.0)	
	3B	17 (7.1)	17 (9.8)	
	3C	29 (12.1)	19 (10.9)	
	4	9 (3.8)	8 (4.6)	
Recurrence	No	229 (97.0)	172 (98.9)	0.312 *
	Yes	7 (3.0)	2 (1.15)	
Metastasis	No	209 (88.6)	164 (94.3)	0.070 ***
	Yes	27 (11.4)	10 (5.8)	
Outcomes	Alive	223 (92.9)	170 (97.7)	0.049 ***
	Dead	17 (7.1)	4 (2.3)	

* Fisher’s exact chi-square test; ** Pearson chi-square test; *** chi-square test with Yates correction. COVID-19: coronavirus-19; PET: positron emission tomography; ASA: American Society of Anesthesiologists; CT: chemotherapy; CRT: chemoradiotherapy; MBR: modified Bloom–Richardson; ER: estrogen receptor; PR: progesterone receptor; TNM: primary tumor size (T), nodal involvement (N), M: metastasis (M).

**Table 4:** Comparison of alive and mortality subgroups in terms of quantitative variables.

Parameters [Median (95%CI)]	Alive (n = 393)	Mortality (n = 21)	*p*
Age (years)	50 (49-52)	54 (51-65)	0.069
From diagnosis to surgery (days)	26 (22-29)	148 (68-229)	0.001
From surgery to pathology report (days)	26 (25-28)	33 (28-41)	0.018
Specimen weight (gram)	882 (824–940)	1414 (947-1876)	0.001
Tubule formation	3 (3-3)	3 (3-3)	0.142
Nuclear pleomorphism	3 (3-3)	3 (3-3)	0.033
Mitotic count	2 (2-3)	3 (3-3)	0.020
Tumor size (mm)	23 (22-25)	42 (36-85)	0.000
Total LAP	10 (9-12)	6 (3-15)	0.609
Positive LAP	0 (0-0)	2 (2-11)	0.032
Ki-67 (%)	20 (20-25)	40 (40-70)	0.009

LAP: lymphadenopathy; Ki-67: proliferation index.

**Table 5:** Comparison of alive and mortality subgroups in terms of qualitative variables.

Parameters	Categories	Alive (n = 393)	Mortality (n = 21)	*p*
Group	Pre-COVID-19	223 (56.7)	17 (81.0)	0.049 ***
	COVID-19 Era	170 (43.3)	4 (19.1)	
Gender	Female	389 (99.0)	21 (100.0)	0.999 *
	Male	4 (1.0)	0 (0.0)	
Comorbidity	No	244 (62.1)	10 (47.6)	0.273 ***
	Yes	149 (37.9)	11 (52.4)	
ASA scores	1	79 (20.1)	4 (19.1)	0.022 **
	2	276 (70.2)	1 (52.4)	
	3	38 (9.7)	6 (28.6)	
Preoperative PET-CT	No	61 (15.5)	2 (9.5)	0.754 *
	Yes	332 (84.5)	19 (90.5)	
Neoadjuvant CT	No	295 (75.1)	8 (38.1)	0.001 ***
	Yes	98 (24.9)	13 (61.9)	
Adjuvant CRT	No	46 (11.7)	7 (33.3)	0.011 *
	Yes	346 (88.3)	14 (66.7)	
Type of surgery	Mastectomy	293 (74.6)	21 (100.0)	0.017 ***
	BCS	100 (25.5)	0 (0.0)	
Which breast	Right	184 (46.8)	13 (61.9)	0.261 ***
	Left	209 (53.2)	8 (38.1)	
Which quadrant	Lower outer	75 (19.1)	5 (23.8)	0.890 **
	Lower inner	45 (11.5)	2 (9.5)	
	Upper outer	176 (44.78)	9 (42.9)	
	Upper inner	83 (21.1)	5 (23.8)	
	Retroareolar	14 (3.6)	1 (0.0)	
Multicentricity	No	353 (89.8)	10 (47.6)	<0.001 *
	Yes	40 (10.2)	11 (52.4)	
Multifocality	No	343 (87.3)	17 (81.0)	0.500 *
	Yes	50 (12.7)	4 (19.1)	
MBR grade	1	38 (11.7)	1 (5.3)	0.110 **
	2	128 (39.4)	4 (21.1)	
	3	159 (48.9)	14 (73.7)	
Skin involvement	No	363 (92.4)	14 (66.7)	0.001 *
	Yes	30 (7.6)	7 (33.3)	
Nipple involvement	No	373 (94.9)	16 (76.2)	0.006 *
	Yes	20 (5.1)	5 (23.8)	
Lymphovascular invasion	No	159 (40.6)	2 (9.5)	0.009 ***
	Yes	233 (59.4)	19 (90.5)	
Perineural invasion	No	306 (78.1)	11 (52.4)	0.014 *
	Yes	86 (21.9)	10 (47.6)	
Comedo necrosis	No	303 (77.1)	16 (76.2)	0.999 *
	Yes	90 (22.9)	5 (23.8)	
ER	Negative	61 (16.0)	6 (28.6)	0.136 *
	Positive	321 (84.0)	15 (71.4)	
PR	Negative	99 (26.3)	10 (52.6)	0.025 ***
	Positive	278 (73.7)	9 (47.4)	
c-erbB2	Negative	261 (69.6)	12 (57.1)	0.338 ***
	Positive	114 (30.4)	9 (42.9)	
E-cadherin	Negative	32 (13.6)	2 (13.3)	0.999 *
	Positive	203 (86.4)	13 (86.7)	
TNM classification	0	18 (4.6)	0 (0.00)	<0.001 **
	IA	80 (20.4)	0 (0.0)	
	IIA	92 (23.4)	5 (23.8)	
	IIB	55 (14.0)	0 (0.0)	
	IIIA	63 (16.0)	2 (9.5)	
	IIIB	32 (8.1)	2 (9.5)	
	IIIC	44 (11.2)	4 (19.1)	
	IV	9 (2.3)	8 (38.1)	
Recurrence	No	385 (98.7)	16 (80.0)	<0.001 *
	Yes	5 (1.3)	4 (20.0)	
Metastasis	No	370 (94.9)	3 (15.0)	<0.001 *
	Yes	20 (5.1)	17 (85.0)	

* Fisher’s exact chi-square test; ** Pearson chi-square test; *** chi-square test with Yates correction COVID: coronavirus-19; PET: positron emission tomography; ASA: American Society of Anesthesiologists; CT: chemotherapy; CRT: chemoradiotherapy; MBR: modified Bloom–Richardson; ER: estrogen receptor; PR: progesterone receptor; TNM: primary tumor size (T), nodal involvement (N), M: metastasis (M).

**Table 6:** Comparison of the survival of Pre-COVID-19 and COVID-19 Era groups.

Groups	Mean (Days)				*p*
	Estimate	Std. Error	95% CI		
			Lower Bound	Upper Bound	
Pre-COVID-19	2151.051	27.690	2096.778	2205.324	0.044
COVID-19 Era	1533.609	15.120	1503.973	1563.245	
Overall	2177.773	19.359	2139.828	2215.717	

## Data Availability

The datasets analyzed during the current study are available from the corresponding authors on reasonable request.
